# Endotoxemia analysis in the postoperative period following cardiac surgery

**DOI:** 10.1186/2197-425X-3-S1-A106

**Published:** 2015-10-01

**Authors:** A Campos Gómez, TM Tomasa Irriguible, ML Cámara Rosell, E Jordana Lluch, J Roca Antonio, S Just Martinez, M Giménez Pérez

**Affiliations:** Hospital Universitario Germans Trías i Pujol, Critical Care, Badalona, Spain; Hospital Universitario Germans Trías i Pujol, Cardiac Surgery, Badalona, Spain; Hospital Universitario Germans Trías i Pujol, Microbiology, Badalona, Spain; Hospital Universitario Germans Trías i Pujol, Epidemiology, Badalona, Spain

## Introduction

Endotoxin is a lipopolysaccharide in the membrane of gram negative bacilli, and is known to be one of the most potent activators of the inflammatory response in humans. In patients subjected to cardiac surgery, transient endotoxemia has been shown in many occasions, which seems to be closely related to extracorporeal circulation. The magnitude of endotoxemia, the high risk criteria and correlation with clinical evolution vary widely between studies.

## Objectives

To examine the prevalence of endotoxemia related to cardiopulmonary bypass (CPB) in a cohort of patients undergoing cardiac surgery, using the Endotoxin Activity Assay (EAA). High risk criteria of endotoxemia were also investigated.

## Methods

EAA assay was performed within two hours of ICU admission in a prospective observational study. An EAA of < 0.40 units was judged as "low”, and ≥0.40 units as "high". Data collected included patient's demographics, cardiac history, EuroSCORE and intra-operative data collected included bypass time and the aortic cross clamp time, drugs and transfusions.

## Results

A total of 107 patients were enrolled. The median age was 66 years (36-87), most were males (69%), 38% had diabetes, 71% hypertension, 12% peripheral vascular disease, 11% chronic renal failure and 21% were active smokers. Median EuroSCORE I was 6 (0-16). Out of 107, 99 were elective, 5 urgent and 3 emergent cardiac surgeries. 73 were valve replacement, 38 coronary vascular diseases, 12 aortic disease and 21 combined surgery. The median CPB time was 95 (24-300) and cross clamp time 68 (17-175) minutes. 37% required blood transfusion. 88% required norepinephrine, 49% dobutamine and 17% epinephrine. Only 24 patients had EAA≥0.4 EA. We found a significant relation between peripheral vascular disease (OR 5,28(1,57-18,42), p = 0,0070) and more red blood packs transfusion with higher EAA levels (OR 1,57 (1,06-3,06), p = 0,06). On the other hand the rest of variables (demographics, cardiac history, EuroSCORE and intra-operative variables) were similar in both groups.

## Conclusions

The results of the study indicate that in postoperative cardiac surgery there is endotoxemia at least in moderate degree. As risk factors, we found that patients with peripheral vascular disease and those who had been transfused with more red blood packs during surgery were at increased risk of endotoxemia.
